# Clonal hematopoiesis as a novel risk factor for type 2 diabetes mellitus in patients with hypercholesterolemia

**DOI:** 10.3389/fpubh.2023.1181879

**Published:** 2023-06-28

**Authors:** Min Joo Kim, Han Song, Youngil Koh, Heesun Lee, Hyo Eun Park, Sung Hee Choi, Ji Won Yoon, Su-Yeon Choi

**Affiliations:** ^1^Department of Internal Medicine, Seoul National University Hospital Healthcare System Gangnam Center, Seoul, Republic of Korea; ^2^Department of Internal Medicine, Seoul National University College of Medicine, Seoul, Republic of Korea; ^3^Genome Opinion Incorporation, Seoul, Republic of Korea; ^4^Department of Internal Medicine, Seoul National University Bundang Hospital, Seongnam, Republic of Korea

**Keywords:** clonal hematopoiesis, diabetes mellitus, hematopoiesis, hypercholesterolemia, LDL cholesterol

## Abstract

**Introduction:**

Clonal hematopoiesis of indeterminate potential (CHIP) is associated with atherosclerosis and cardiovascular disease. It has been suggested that CHIP may be related to diabetes, so we investigated the association between CHIP and new-onset type 2 diabetes.

**Methods:**

This study included 4,047 subjects aged >=40 years without diabetes. To detect CHIP, targeted gene sequencing of genomic DNA from peripheral blood cells was performed. The incidence of new-onset type 2 diabetes during the follow-up period was evaluated.

**Results:**

Of the total subjects, 635 (15.7%) had CHIP. During the median follow-up of 5.1 years, the incidence of new-onset diabetes was significantly higher in CHIP carriers than in subjects without CHIP (11.8% vs. 9.1%, *p* = 0.039). In a univariate analysis, CHIP significantly increased the risk of new-onset diabetes (HR 1.32, 95% CI 1.02–1.70, *p* = 0.034), but in a multivariate analysis, it was not significant. The CHIP-related risk of new onset diabetes differed according to LDL cholesterol level. In the hyper-LDL cholesterolemia group, CHIP significantly increased the risk of diabetes (HR 1.64, 95% CI 1.09–2.47, *p* = 0.018), but it did not increase the risk in the non-hyper-LDL cholesterolemia group. The subjects with CHIP and hyper-LDL-cholesterolemia had approximately twice the risk of diabetes than subjects without CHIP and with low LDL cholesterol (HR 2.05, 95% CI 1.40–3.00, *p* < 0.001).

**Conclusion:**

The presence of CHIP was a significant risk factor for new-onset type 2 diabetes, especially in subjects with high LDL cholesterol. These results show the synergism between CHIP and high LDL cholesterol as a high-risk factor for diabetes.

## 1. Introduction

The development of type 2 diabetes is very complex, involving several genetic and environmental factors. Age, race/ethnicity, family history of diabetes, obesity, and physical inactivity are known as traditional risk factors (1). However, these traditional risk factors alone cannot fully explain the development of type 2 diabetes. The previous prediction models generated with these traditional risk factors had limited ability to predict future type 2 diabetes (2–4). Therefore, studies have been conducted to find new factors conferring residual risk for type 2 diabetes. For example, genome-wide association studies have recently been performed to find new genetic risk factors, and based on these studies, it is estimated that genetic variation can explain 18%−45% of the risk for type 2 diabetes (5, 6). Even so, there are still risk factors for type 2 diabetes that have not been discovered.

Aging is accompanied by the accumulation of somatic mutations in hematopoietic stem cells. These mutations can cause hematologic malignancies, such as acute leukemia, but this does not happen in most individuals. The clonal expansion of hematopoietic cells with aging-related recurrent somatic mutations in the absence of other hematologic abnormalities is called clonal hematopoiesis of indeterminate potential (CHIP) (7). CHIP has been reported to be associated with an increased risk of cardiovascular disease, including coronary heart disease and stroke, and higher mortality (8, 9). CHIP is an emerging risk factor for atherosclerosis (10, 11).

Type 2 diabetes is a potent risk factor for cardiovascular disease and atherosclerosis. Like CHIP, type 2 diabetes is associated with aging. A meta-analysis reported that type 2 diabetes is associated with an increased risk of hematologic malignancy (12). These results suggest that CHIP may have some relationship with type 2 diabetes. Bonnefond et al. reported that large clonal mosaicism in peripheral blood cells was associated with type 2 diabetes (13). Jaiswal et al. reported that CHIP was modestly but significantly associated with an increased risk of type 2 diabetes (odds ratio 1.3) (14). Owing to the limitations of cross-sectional studies, whether CHIP promotes the development of type 2 diabetes is not yet understood. Therefore, in this study, we investigated the association between CHIP and new-onset type 2 diabetes in a longitudinal retrospective cohort study.

## 2. Materials and methods

### 2.1. Study population

The Gene-ENvironmental Interaction and phEnotype (GENIE)-CHIP cohort was designed to investigate the effects of CHIP on health outcomes. This retrospective cohort included subjects aged ≥65 years or aged 40–64 years with more than one of the risk factors for cardiovascular disease (diabetes, hypertension, dyslipidemia, chronic kidney disease, and current smoking) who came to the Seoul National University Hospital Healthcare System Gangnam Center for a health check-up for screening purposes between January 2014 and January 2017. The GENIE-CHIP cohort was a subcohort of the GENIE cohort, and details of the GENIE cohort were described previously (15). Of the total 4,991 subjects in the GENIE-CHIP cohort, subjects who did not have glucose or low-density lipoprotein (LDL) cholesterol (*n* = 64) were excluded, and 4,927 subjects were finally analyzed (Supplementary Figure S1). Type 2 diabetes was defined as a fasting glucose ≥126 mg/dl or HbA1c ≥6.5% and/or treatment with glucose-lowering medication. Hypertension was defined as a systolic blood pressure (BP) ≥140 mmHg or diastolic BP ≥90 mmHg or the use of anti-hypertensive medications. Dyslipidemia was defined as total cholesterol ≥240 mg/dL, LDL cholesterol ≥160 mg/dL, triglycerides ≥200 mg/dL, high-density lipoprotein (HDL) cholesterol < 40 mg/dL, or the use of medication for dyslipidemia.

Informed consent was obtained from participants in the GENIE cohort, and blood samples were collected with the approval of the Seoul National University Hospital Institutional Review Board (H-1103-127-357). The protocol of this retrospective cohort study was additionally approved by the institutional review board (H-1908-121-1056), and informed consent was waived owing to its retrospective nature.

### 2.2. Targeted gene sequencing

Genomic DNA was isolated from peripheral blood. Targeted gene sequencing was performed using an Agilent SureSelectXT HS custom panel and the Illumina NovaSeq 6000 with 2 × 150 bp paired-end reads with a minimum coverage of 800 ×. The custom panel comprised all the exons of 89 genes frequently involved in CHIP, such as DNMT3A, TET2, ASXL1, JAK2, and TP53 (8, 14, 16–18). The detailed process of CHIP variant calling is described in a previous study (19). The unreliable variants, which met any one of the following criteria, were filtered out as sequencing artifacts or germline variants as follows: (1) the number of altered reads on the positive and negative strands was < 5, the mapping quality was < 30, or the base quality was < 30; (2) the variant allele frequency (VAF) was not between 1.5% and 30%; (3) it was among the common germline variants, including those listed in genomAD, 1 k Genome v3, ESP6500, and ExAC; and (4) it was listed in the artifact database with a minor allele frequency of >2% in the internal panel of 1,000 Korean individuals. All reliable non-synonymous variants were annotated as CHIP mutations.

### 2.3. Statistical analyses

Continuous variables are presented as the mean ± standard deviation or median with interquartile range and were compared using Student's *t*-test for independent samples or the Mann–Whitney test. Categorical variables are expressed as numbers and percentages and were compared using the χ2 test or Fisher's exact test. The association between the presence of CHIP and new-onset type 2 diabetes was tested using a Cox proportional hazard model with covariates including age, sex, BMI, and other diabetes risk factors. Diabetes-free survival, estimated using the Kaplan–Meier method, was compared between the groups using the log-rank test. The attributable proportion (AP) due to the interaction between CHIP and high LDL cholesterol was calculated using the epiR package of R statistics (version 4.1.2., R Foundation for Statistical Computing). For this analysis, participants were included in the hyper-LDL cholesterolemia (hyperLDLC) group if their LDL cholesterol level was ≥160 mg/dL (classified as “high level” by the National Cholesterol Education Program) (20, 21) or if they took medication for dyslipidemia. Samples with covariates missing were removed in the risk analysis and interaction analyses. All statistical analyses were performed using Python version 3.7.9 (Python Software Foundation) and its packages Numpy (1.19.4), Scipy (1.5.3), Scikit-learn (0.23.2), and Lifelines (0.25.6).

## 3. Results

### 3.1. Baseline characteristics and the prevalence of type 2 diabetes

Of the total 4,927 subjects, the mean age was 55.4 ± 8.1 years, 73% were men, and 790 (16.0%) had CHIP (Supplementary Table S1). The prevalence of subjects with CHIP (CHIP carrier) increased with age, and gene mutations in DNMT3A and TET2 were the most frequently identified (Supplementary Figure S2). Compared with subjects without CHIP, CHIP carriers were significantly older (58.7 ± 8.5 years old vs. 54.7 ± 7.9 years old, *p* < 0.001) and had lower levels of LDL cholesterol (120.9 ± 31.7 mg/dL vs. 125.4 ± 32.8 mg/dL, *p* < 0.001). However, sex, BMI, waist circumference, and the prevalence of hypertension and dyslipidemia were not different between subjects with and without CHIP (Supplementary Table S1). Of the total 4,927 subjects, 696 (14.1%) had type 2 diabetes. The prevalence of type 2 diabetes in subjects with CHIP (15.2%) was higher than that in subjects without CHIP (13.9%), but it was statistically insignificant.

### 3.2. Impact of CHIP on the incidence of new-onset type 2 diabetes

**Figure 1:**
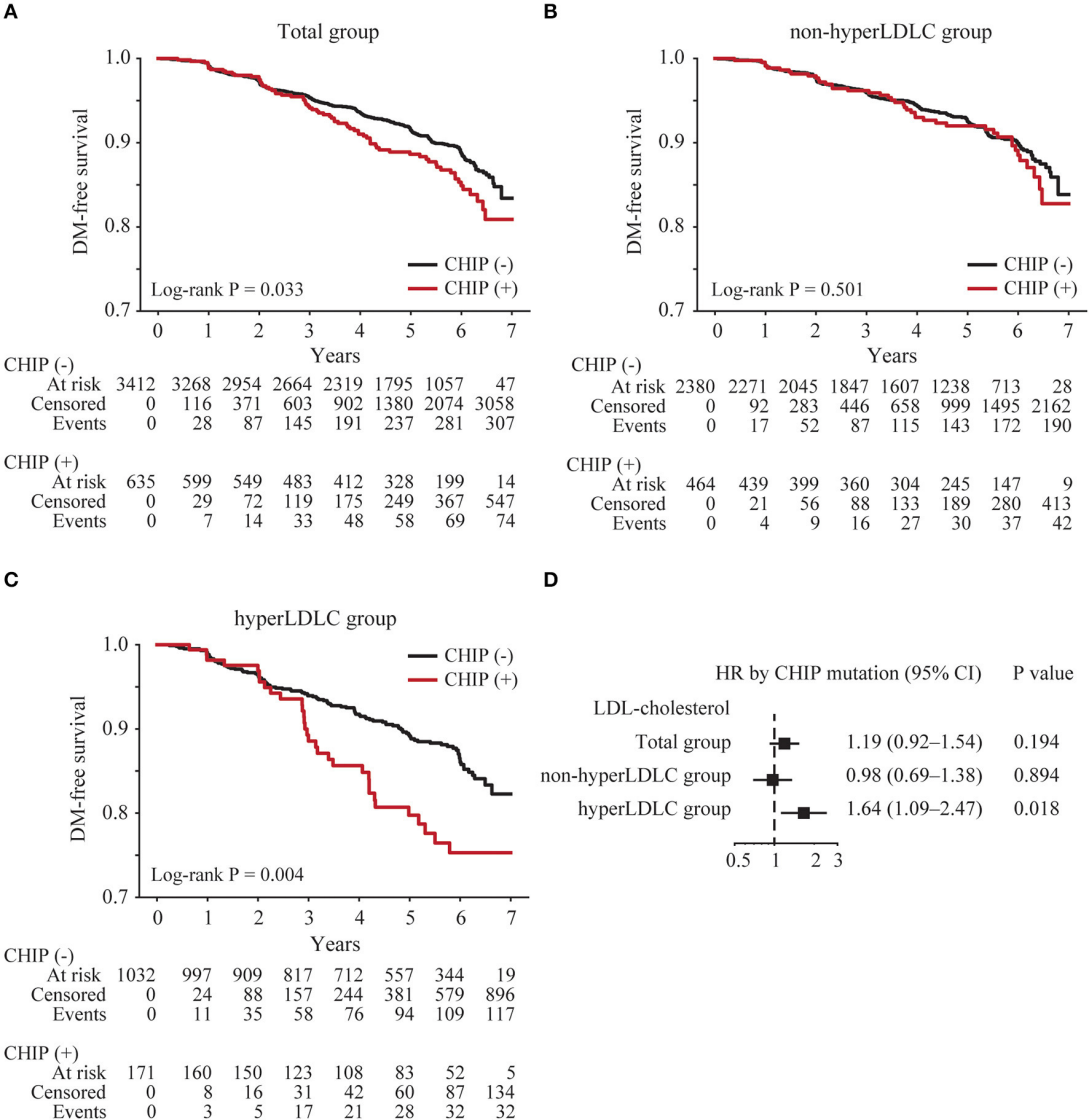
Risk of new-onset type 2 diabetes according to the presence of CHIP. Kaplan–Meier curves were plotted for all subjects (total group) (*n* = 4,047) **(A)** subjects with low LDL cholesterol levels (non-hyperLDLC group) **(B)**, and subjects with high LDL cholesterol levels (hyperLDLC group) **(C)**. The multivariate Cox regression model was analyzed adjusting for age, sex, BMI, and family history of diabetes in the total group, non-hyperLDLC group, and hyperLDLC group **(D)**.

Among the 4,231 subjects without type 2 diabetes, 4,047 (96%) had at least one follow-up examination. The baseline blood levels of glucose and HbA1C were slightly higher in subjects with CHIP than in subjects without CHIP (Table 1). The median follow-up duration was 5.1 years (interquartile range 3.3–6.1 years), and 385 (9.5%) subjects were newly diagnosed with type 2 diabetes. The median time to new-onset type 2 diabetes was 3.1 years (interquartile range 2.0–4.9 years). The incidence of type 2 diabetes in CHIP carriers (11.8%) was significantly higher than that in subjects without CHIP (9.1%) (*p* = 0.039). The Kaplan–Meier curve demonstrated that CHIP carriers had a higher risk of new-onset type 2 diabetes (log-rank *p* = 0.033; Figure 1A). To investigate the effect of CHIP on new-onset type 2 diabetes, a Cox regression analysis was performed. In a univariate analysis, CHIP significantly increased the risk of new-onset type 2 diabetes (hazard ratio [HR] 1.32, 95% confidence interval [CI] 1.02–1.70, *p* = 0.034). However, in a multivariate analysis adjusting for age, sex, BMI, and family history of diabetes, it was not significant (HR 1.19, 95% CI 0.92–1.54, *p* = 0.194) (Supplementary Table S2).

**Table 1:** Clinical characteristics of study subjects according to CHIP status.

	**Total (*n* = 4,047)**	**CHIP carrier (*n* = 635)**	**Non-carrier (*n* = 3,412)**	***p***
Age, years	54.8 ± 8.0	58.3 ± 8.6	54.1 ± 7.7	< 0.001
Male	2,915 (72.0)	454 (71.5)	2,461 (72.1)	1.000
BMI, kg/m^2^	24.0 ± 2.8	24.0 ± 2.5	24.0 ± 2.8	0.793
BMI ≥ 25 kg/m^2^	1,356 (33.5)	224 (35.3)	1,132 (33.2)	0.314
WC, cm	85.5 ± 7.7	86.0 ± 7.3	85.4 ± 7.7	0.096
Systolic BP, mmHg	118.2 ± 13.2	118.8 ± 13.2	118.1 ± 13.2	0.263
Diastolic BP, mmHg	78.6 ± 10.1	77.9 ± 9.5	78.7 ± 10.2	0.071
Fasting glucose, mg/dL	98.0 ± 9.5	98.7 ± 9.8	97.9 ± 9.5	0.060
HbA1C, %	5.6 ± 0.3	5.7 ± 0.3	5.6 ± 0.3	0.005
Total cholesterol, mg/dL	198.9 ± 36.9	194.7 ± 38.9	199.6 ± 36.5	0.002
Triglycerides, mg/dL	106.0 (74.0–150.0)	102.0 (73.0–145.0)	107.0 (75.0–151.0)	0.067
HDL cholesterol, mg/dL	52.0 ± 12.4	51.8 ± 12.3	52.0 ± 12.4	0.728
LDL cholesterol, mg/dL	127.3 ± 32.0	123.5 ± 31.1	128.0 ± 32.1	0.001
Hypertension	1,425 (35.2)	226 (35.6)	1,199 (35.1)	0.821
Dyslipidemia	2,178 (53.8)	323 (50.9)	1,855 (54.4)	0.109
Medication for dyslipidemia	540 (13.3)	93 (14.6)	447 (13.1)	0.309
Family history of diabetes	980 (24.2)	148 (23.3)	832 (24.4)	0.579
Follow-up duration, years	5.1 (3.3–6.1)	5.1 (3.0–6.1)	5.1 (3.3–6.1)	0.323
New-onset type 2 diabetes	385 (9.5)	75 (11.8)	310 (9.1)	0.039

Values are mean ± standard deviation, median (interquartile range), or *n* (%).

CHIP, clonal hematopoiesis of indeterminate potential; BMI, body mass index; WC, waist circumference; BP, blood pressure.

In addition, it was investigated whether CHIP clone size or specific CHIP mutation contributed more to the increased risk of new-onset type 2 diabetes. Among CHIP carriers, 14.4% (92/635) had large CHIP clones defined as VAF ≥10%. Large CHIP carriers had a higher risk of new-onset type 2 diabetes compared with small CHIP carriers or subjects without CHIP in the Kaplan–Meier curve (Supplementary Figure S3). When examining individual CHIP mutations, there was no single mutation that significantly increased the risk of new-onset type 2 diabetes (Supplementary Figure S3).

### 3.3. Impact of CHIP on new-onset type 2 diabetes and its interaction with LDL cholesterol

To identify any subgroups that had a greater impact on CHIP, an interaction analysis between CHIP and clinical factors was conducted. Among the clinical factors, only LDL cholesterol showed a significant interaction with CHIP for new-onset type 2 diabetes (*p* = 0.030, Supplementary Table S3). Therefore, the subjects were divided into the non-hyperLDLC group (LDL cholesterol < 160 mg/dL and no medication for dyslipidemia) and the hyperLDLC group (LDL cholesterol ≥160 mg/dL and/or medication for dyslipidemia). The Kaplan–Meier curve demonstrated that CHIP carriers had a higher risk of new-onset type 2 diabetes only in the hyperLDLC group (log-rank *p* = 0.004; Figures 1B, C). A multivariate Cox regression analysis also showed that CHIP significantly increased the risk of new-onset type 2 diabetes in the hyperLDLC group (adjusted HR 1.64, 95% CI 1.09–2.47, *p* = 0.018) but not in the non-hyperLDLC group (adjusted HR 0.98, 95% CI 0.69–1.38, *p* = 0.894) (Figure 1D). Furthermore, CHIP and high LDL cholesterol demonstrated a significant synergistic effect on the development of new-onset type 2 diabetes (Table 2). The subgroup analysis showed that CHIP carriers in the hyperLDLC group had an ~2-fold higher risk of developing new-onset type 2 diabetes than non-CHIP carriers in the non-hyperLDLC group (HR 2.05, 95% CI 1.40–3.00, *p* < 0.001). This interaction between LDL cholesterol and CHIP may significantly contribute to the development of new-onset type 2 diabetes, at a rate of 34% (Supplementary Table S3). It suggested the synergistic effect of CHIP and high LDL cholesterol on new-onset type 2 diabetes. To exclude the effects of statins on the risk of new-onset type 2 diabetes, a subgroup analysis was conducted only in those who did not take medication for dyslipidemia, and the results were similar (Supplementary Table S4).

**Table 2:** Subgroup analysis of the risk of new-onset type 2 diabetes based on CHIP status and LDL cholesterol level.

	**New-onset type 2 diabetes (*n* = 377)**	**No event (*n* = 3,644)**	**Adjusted HR (95% CI)**	***p***
Non-hyperLDLC group and non-carrier	188 (49.9)	2184 (59.9)	1.00 (reference)	
Non-hyperLDLC group and CHIP carrier	41 (10.9)	414 (11.4)	1.03 (0.73–1.45)	0.880
HyperLDLC group and non-carrier	116 (30.8)	908 (24.9)	1.32 (1.05–1.67)	0.020
HyperLDLC group and CHIP carrier	32 (8.5)	138 (3.8)	2.05 (1.40–3.00)	< 0.001

CHIP, clonal hematopoiesis of indeterminate potential; HR, hazard ratio; CI, confidence interval.

## 4. Discussion

The present study demonstrated that CHIP increased the risk of new-onset type 2 diabetes in subjects with high LDL cholesterol. CHIP had a significant interaction with LDL cholesterol, and they showed synergism in increasing the risk for new-onset type 2 diabetes. The risk for new-onset type 2 diabetes in CHIP carriers in the hyperLDLC group was twice that of non-CHIP carriers in the non-hyperLDLC group.

### 4.1. Association between CHIP and type 2 diabetes

To the best of our knowledge, this is the first cohort study demonstrating a significant association between CHIP and the development of new-onset type 2 diabetes. Although Jaiswal et al. reported the association between CHIP and type 2 diabetes for the first time, it was a cross-sectional study (14). Additionally, its association was only significant in European and South Asian populations (14). In the present study of an East Asian population, the prevalence of type 2 diabetes was not different between subjects with and without CHIP, but CHIP increased the risk of new-onset type 2 diabetes. In this study, we accepted a broader definition of CHIP mutations than the previous study (14). CHIP mutations are classified into putative drivers (PD-CHIP) and non-putative drivers (non-PD-CHIP) (13). While Jaiswal et al. defined only hematologic cancer driver mutations as CHIP mutations (PD-CHIP), we included both PD and non-PD-CHIP mutations that cause any of the non-synonymous amino acid alterations in this study. Clonal expansions can be induced by various genetic alterations, including copy number variants (CNVs), structural variations, and epigenetic changes, not just somatic “driver” mutations (PD-CHIP). Hence, non-PD-CHIP mutations could be considered “passengers” in clonal expansion caused by other alterations that could not be detected using our targeted panels but may still have clinical consequences. Recently, it is reported that a large CHIP clone defined as VAF ≥10% was associated with a greater risk of cardiovascular disease and all-cause mortality (22). In this study, the Kaplan–Meier curve showed that large CHIP was associated with a higher risk of new-onset type 2 diabetes compared with small CHIP (Supplementary Figure S3). However, it was insignificant in a multivariate analysis adjusting for age, sex, BMI, and family history of diabetes (HR 1.62, 95% CI 0.96–2.72, *p* = 0.069), and this may be because of the small number of large CHIP carriers. Further large-scale longitudinal studies on the association between CHIP and new-onset type 2 diabetes are needed to validate our results.

### 4.2. Plausible mechanism underlying the effect of CHIP on the development of type 2 diabetes

Among genes mutated in CHIP, DNMT3A and TET2 were the most frequent (9, 14, 23). Consistent with these results, DNMT3A and TET2 were the most commonly mutated genes in both our total subjects and our subjects with new-onset type 2 diabetes (Supplementary Figure S2).

CHIP may affect insulin resistance and contribute to the development of type 2 diabetes. Fuster et al. generated mice with CHIP to investigate the effect of CHIP on atherosclerosis and insulin resistance (24, 25). Tet2 loss-of-function-driven clonal hematopoiesis showed a progressive aggravation of insulin resistance and an increase in fasting blood glucose levels in aged, obese mice (25). It was accompanied by increased proinflammatory cytokine interleukin (IL)-1β expression in white adipose tissues. These results suggest that inflammation is an important mechanism underlying the effect of CHIP on insulin resistance.

CHIP shifts macrophages to a more inflammatory state. Dnmt3a or Tet2-deficient J774.1 myeloid cells created using a lentivirus/CRISPR system showed increased expression of inflammatory cytokines and chemokines, such as IL-1β, IL-6, and CCL5 (26). Inflammatory cytokines, including IL-1β, were upregulated in macrophages isolated from mice with Tet2 deficiency restricted to myeloid cells (Mye-Tet2-KO mice) compared with macrophages from control mice (24). In patients with heart failure, peripheral blood monocytes of patients carrying DNMT3A mutations have demonstrated a significant upregulation of inflammatory genes compared with those of patients without DNMT3A mutations (27). Type 2 diabetes is a chronic inflammatory disease (28). The infiltration and expansion of macrophage and the production of proinflammatory cytokines in several tissues, including the pancreatic islets, adipose tissue, muscle, and liver, contribute to the development of type 2 diabetes. Therefore, more inflammatory macrophages related to CHIP may be an underlying mechanism of the increased risk of new-onset type 2 diabetes.

### 4.3. The effect of the interaction between CHIP and high LDL cholesterol levels on the development of new-onset type 2 diabetes

In this study, the interaction between CHIP and high LDL cholesterol levels showed a synergistic effect on the development of new-onset type 2 diabetes. CHIP significantly increased the risk of new-onset type 2 diabetes in the hyperLDLC group (adjusted HR 1.64) but not in the non-hyperLDLC group (adjusted HR 0.98). The hyperLDLC group included 45% (540/1203) statin users. Statin use can increase the risk of diabetes, and a meta-analysis reported an odds ratio of 1.09 for new-onset type 2 diabetes (29, 30). Even after excluding statin users, CHIP still increased the risk of new-onset type 2 diabetes (Supplementary Table S4). This result suggests that the synergistic effect of CHIP and LDL cholesterol on the development of new-onset type 2 diabetes was independent of statin use. The underlying hypothesis for the synergism of CHIP and high LDL cholesterol is complex. First, LDL cholesterol itself may have exacerbated inflammation (31), leading to the development of type 2 diabetes. In particular, oxidized LDL and small dense LDL are more proinflammatory and proatherogenic than naïve LDL (32, 33), and they have also been reported to be associated with new-onset type 2 diabetes (34). Oxidized LDL cholesterol activates the nucleotide-binding domain, leucine-rich-containing family, and pyrin domain-containing-3 (NLRP3) inflammasome in the cytoplasm of macrophages, and NLRP3 inflammasome leads to proinflammatory cytokines IL-1β release (35). Therefore, LDL cholesterol and CHIP may boost macrophage activation through the inflammasome, resulting in a synergistic effect on the development of type 2 diabetes. Second, high LDL cholesterol levels may stimulate the proliferation of hematopoietic stem cells with CHIP mutations and increase the number of inflammatory myeloid cells in peripheral blood. Increased cholesterol levels can promote the proliferation and mobilization of hematopoietic stem cells (36, 37). APOE-deficient (Apoe^−/−^) or LDL receptor-deficient (Ldlr^−/−^) mice fed high-fat diets showed proliferation of hematopoietic stem cells as well as leukocytosis in peripheral blood (38–40). These results suggest that high LDL cholesterol levels may potentiate the effects of CHIP on type 2 diabetes.

### 4.4. Limitations

This study has some limitations. Since our study did not evaluate inflammatory markers/cytokines, such as IL-6, oxidized LDL, small dense LDL, or insulin resistance, it was difficult to elucidate the mechanism by which CHIP interacts with LDL cholesterol to increase the risk of type 2 diabetes. Further studies are needed to determine the underlying mechanism of the association between CHIP and type 2 diabetes. The time and number of follow-up examinations were different for each subject due to the nature of this retrospective cohort study.

## 5. Conclusion

This is the first study to demonstrate that the presence of CHIP was significantly associated with the development of type 2 diabetes. In particular, CHIP and high LDL cholesterol levels synergistically increased the risk of new-onset type 2 diabetes. CHIP may be a hidden risk factor for type 2 diabetes.

## Data availability statement

The datasets presented in this article are not readily available because of patent issues. Requests to access the datasets should be directed to HS (shany00@gmail.com).

## Ethics statement

The studies involving human participants were reviewed and approved by Seoul National University Hospital Institutional Review Board. The patients/participants provided their written informed consent to participate in this study.

## Author contributions

YK and S-YC designed the study. MK, HS, YK, HL, HP, JY, and S-YC collected, analyzed, and interpreted the data. MK and HS wrote the manuscript. YK, HL, HP, SC, JY, and S-YC contributed to the discussion and reviewed and edited the manuscript. All authors contributed to the article and approved the submitted version.
