# Supplementary material for: Clonal hematopoiesis as a novel risk factor for type 2 diabetes mellitus in patients with hypercholesterolemia
Source: Front Public Health. 2023 Jun 28;11:1181879. doi: 10.3389/fpubh.2023.1181879 (PMC10345505; doi:10.3389/fpubh.2023.1181879)
Supplement: Supplementary file 1 [file Table_1.docx]

Supplementary Material

Clonal Hematopoiesis as a Novel Risk Factor for Type 2 Diabetes Mellitus in Patients with Hypercholesterolemia

Min Joo Kim^†^, Han Song^†^, Youngil Koh, Heesun Lee, Hyo Eun Park, Sung Hee Choi, Ji Won Yoon^*^, Su-Yeon Choi^*^

*** Correspondence:** Ji Won Yoon: jwyoonmd@gmail.com and Su-Yeon Choi: sychoi9@gmail.com

# Supplementary Figures and Tables

## Supplementary Figures

**Supplementary Figure S1.** Flow diagram of this study.


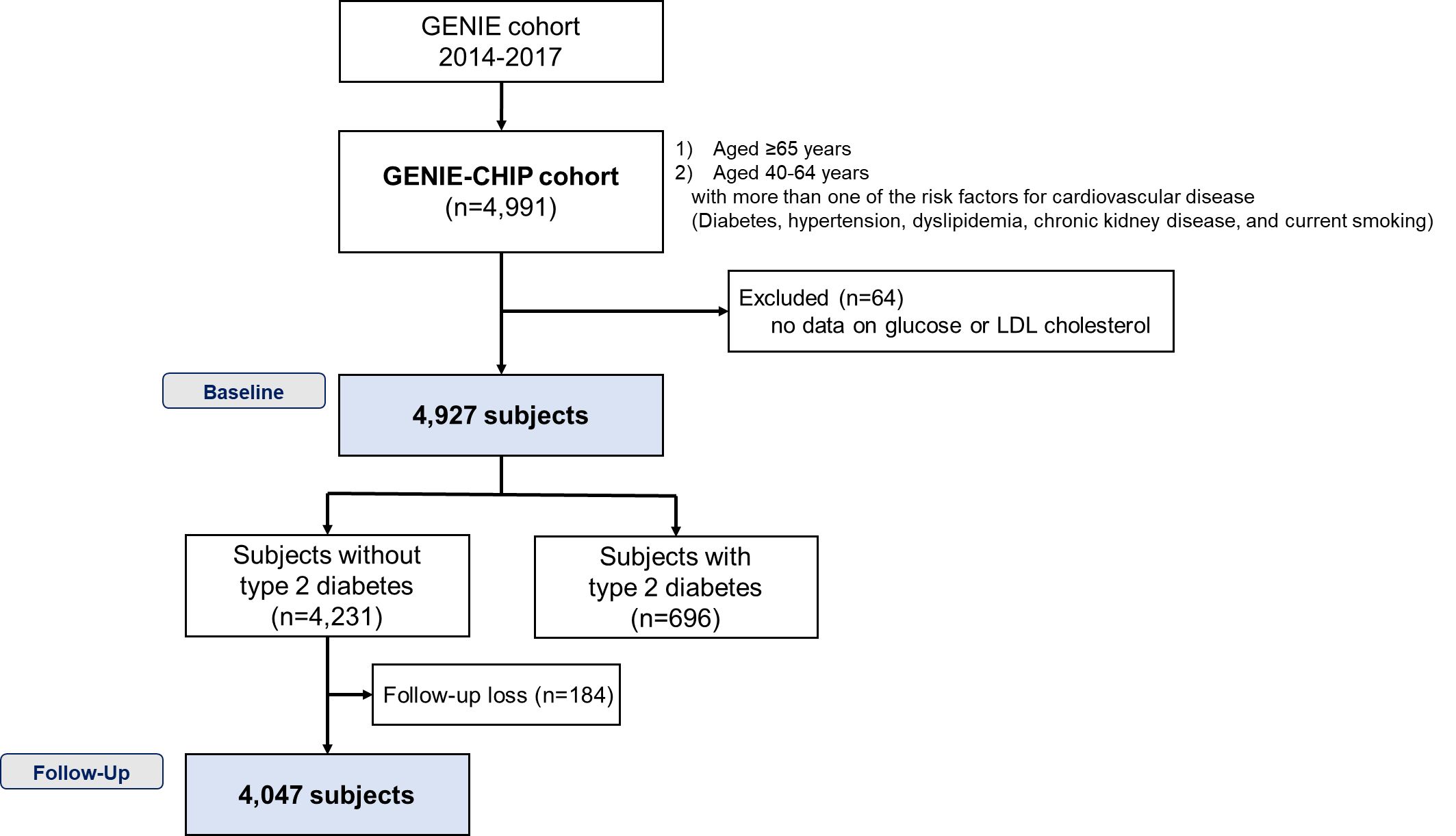


**Supplementary Figure S2.** Clinical genetic characteristics of CHIP. (A) The most common mutations and its proportion in total CHIP carriers and CHIP carriers with type 2 diabetes (B) The prevalence of CHIP according to the age.
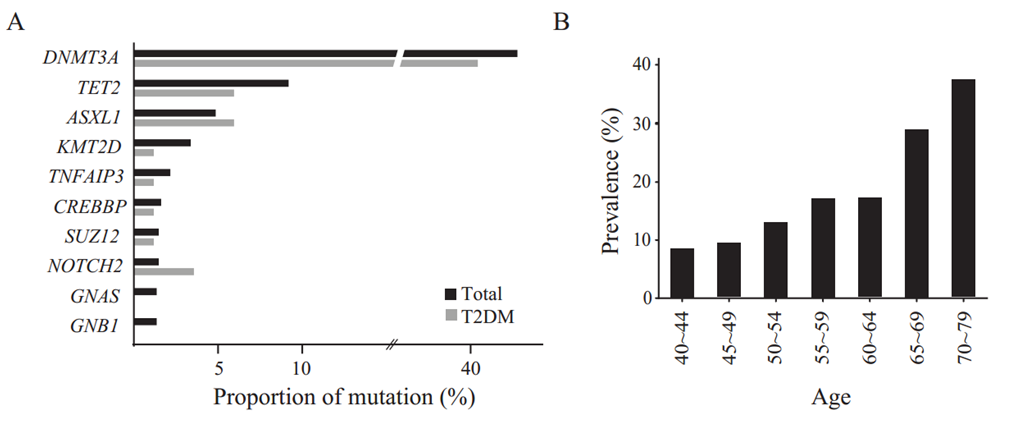


**Supplementary Figure S3.** Risk of new-onset type 2 diabetes according to the CHIP clone size and CHIP mutation. Kaplan–Meier curves were plotted to compare subjects with large CHIP, small CHIP, and without CHIP (A), subjects with and without *DNMT3A* mutation (B), subjects with *TET2* mutation (C), subjects with *ASXL1* mutation (D).


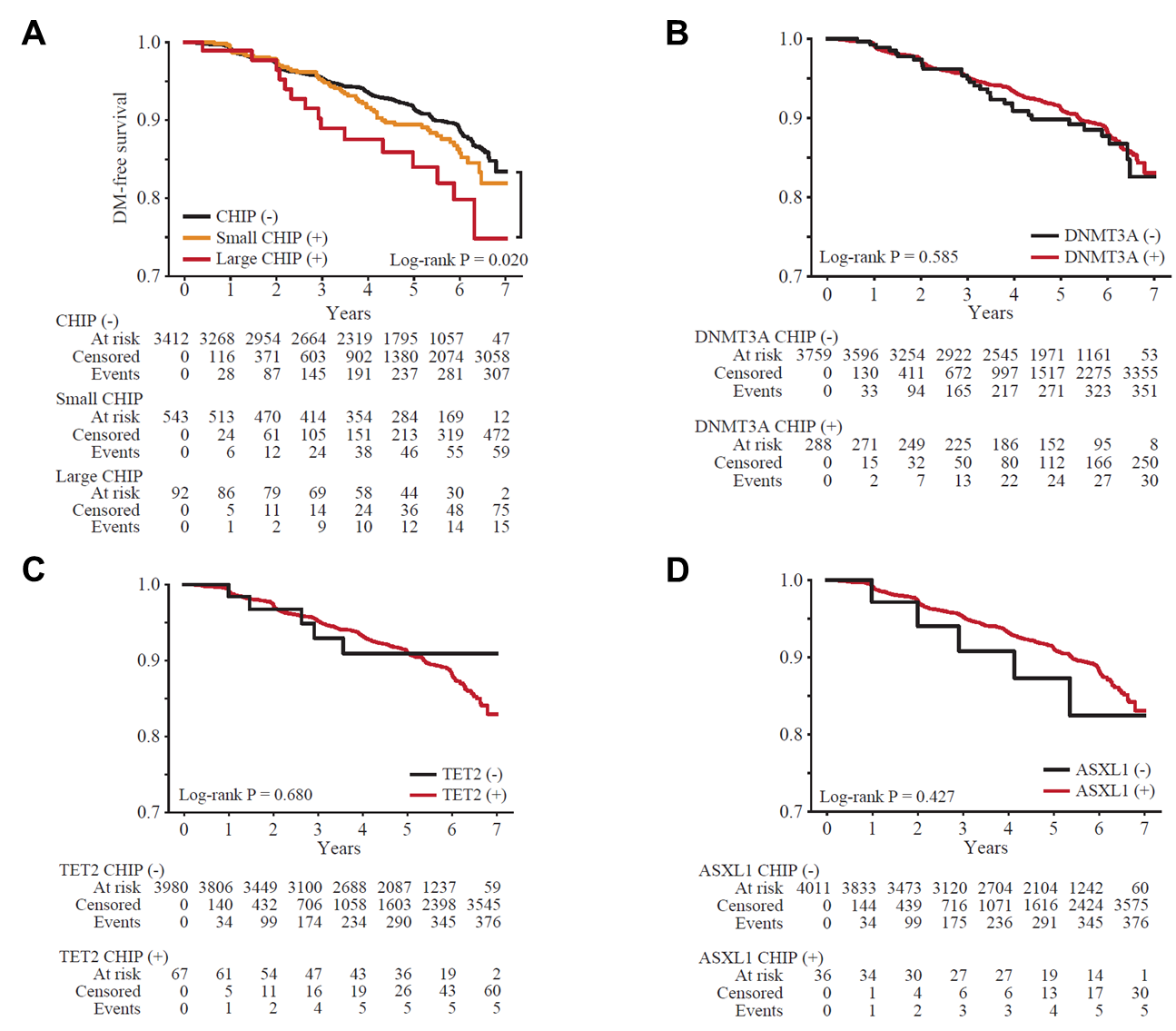


## Supplementary Tables

**Supplementary Table S1**

Clinical characteristics of study subjects according to the CHIP status.

|  | Total  (n = 4,927) | CHIP carrier  (n = 790) | Non-carrier  (n = 4,137) | *p* |
| --- | --- | --- | --- | --- |
| Age, years | 55.4±8.1 | 58.7±8.5 | 54.7±7.9 | <0.001 |
| Male, % | 3,598 (73.0) | 596 (72.3) | 3,002 (73.2) | 1.000 |
| Smoking |  |  |  | <0.001 |
| Current smoker | 1,181 (24.0) | 153 (19.4) | 1,028 (24.8) |  |
| Former smoker | 1,553 (31.5) | 296 (37.5) | 1,257 (30.4) |  |
| Never smoker | 2,193 (44.5) | 341 (43.2) | 1,852 (44.8) |  |
| BMI, kg/m^2^ | 24.1±2.8 | 24.1±2.5 | 24.1±2.9 | 0.739 |
| BMI ≥25kg/m^2^ | 1,749 (35.5) | 279 (35.3) | 1,470 (35.5) | 0.935 |
| WC, cm | 86.0±7.8 | 86.3±7.2 | 85.9±7.9 | 0.221 |
| Systolic BP, mmHg | 118.7±13.2 | 119.5±13.3 | 118.5±13.2 | 0.055 |
| Diastolic BP, mmHg | 78.8±10.0 | 78.2±9.4 | 78.9±10.1 | 0.067 |
| Fasting glucose, mg/dl | 103.7±20.7 | 104.9±20.5 | 103.5±20.7 | 0.086 |
| HbA1C, % | 5.8±0.7 | 5.9±0.7 | 5.8±0.7 | 0.046 |
| Total cholesterol, mg/dl | 195.8±37.8 | 191.6±38.9 | 196.6±37.5 | <0.001 |
| Triglycerides, mg/dl | 107.0 (76.0–153.0) | 103.5 (74.0–148.8) | 108.0 (76.0–154.0) | 0.095 |
| HDL cholesterol, mg/dl | 51.6±12.2 | 51.5±11.9 | 51.6±12.2 | 0.805 |
| LDL cholesterol, mg/dl | 124.7±32.7 | 120.9±31.7 | 125.4±32.8 | <0.001 |
| Hypertension | 1,834 (37.2) | 304 (38.5) | 1,530 (37.0) | 0.422 |
| Type 2 diabetes | 696 (14.1) | 120 (15.2) | 576 (13.9) | 0.344 |
| Dyslipidemia | 2,652 (53.8) | 406 (51.4) | 2,246 (54.3) | 0.139 |
| Medication for dyslipidemia | 730 (14.8) | 131 (16.6) | 599 (14.5) | 0.126 |

**Supplementary Table S2**

Risk factors for new-onset type 2 diabetes.

|  | Adjusted HR (95% CI) | *p* |
| --- | --- | --- |
| CHIP mutation | 1.19 (0.92–1.54) | 0.194 |
| Age (per 1 year increment) | 1.03 (1.02–1.04) | <0.001 |
| Male | 1.07 (0.84–1.37) | 0.575 |
| BMI (per 1 kg/m^2^ increment) | 1.18 (1.14–1.22) | <0.001 |
| Family history of diabetes | 1.62 (1.31–2.00) | <0.001 |
| Hypertension | 1.50 (1.22–1.85) | <0.001 |
| High triglycerides | 1.66 (1.28–2.16) | <0.001 |
| Low HDL cholesterol | 0.95 (0.71–1.27) | 0.716 |
| High LDL cholesterol | 1.43 (1.16–1.76) | <0.001 |

HR, hazard ratio; CI, confidence interval; CHIP, clonal hematopoiesis of indeterminate potential; BMI, body mass index.

**Supplementary Table S3**

Interaction between CHIP status and clinical factors on new-onset type 2 diabetes (n = 4,021).

|  | CHIP carrier | | CHIP non-carrier | | Attributable proportion, % (95% CI) | *p* |
| --- | --- | --- | --- | --- | --- | --- |
| Clinical factor | No | Yes | No | Yes |  |  |
| Age ≥ 60years | 39/383 (10.2%) | 34/242 (14.0%) | 220/2661 (8.3%) | 84/735 (11.4%) | 5.4 (-37.0–47.8) | 0.815 |
| Male | 20/178 (11.2%) | 53/447 (11.9%) | 67/944 (7.1%) | 237/2452 (9.7%) | -21.8 (-54.9–41.4) | 0.509 |
| Obesity (BMI ≥25kg/m^2^) | 36/401 (9.0%) | 37/224 (16.5%) | 153/2264 (6.8%) | 151/1132 (13.3%) | -8.2 (-51.6–35.1) | 0.722 |
| Family history of diabetes | 45/481 (9.4%) | 28/144 (19.4%) | 204/2565 (8.0%) | 100/831 (12.0%) | 29.3 (-3.9–62.4) | 0.083 |
| Hypertension | 35/405 (8.6%) | 38/220 (17.3%) | 154/2200 (7.0%) | 150/1195 (12.6%) | 12.3 (-25.6–50.3) | 0.535 |
| High triglycerides | 60/553 (10.8%) | 13/72 (18.1%) | 243/2964 (8.2%) | 61/432 (14.1%) | -1.2 (-63.4–61) | 0.973 |
| Low HDL cholesterol | 57/530 (10.8%) | 16/95 (16.8%) | 265/2933 (9.0%) | 39/463 (8.4%) | 38.2 (-2.4–78.8) | 0.065 |
| High LDL cholesterol | 41/455 (9.0%) | 32/170 (18.8%) | 188/2372 (7.9%) | 116/1024 (11.3%) | 34.1 (3.2–65.0) | 0.030 |

Values are n of event/total subjects (%).

Attributable proportion due to interaction was calculated from (RR_11_-RR_10_-RR_01_+1)/RR_11_. RR_AB_ is the relative risk of disease if both factor A and B are present (1) or absence (0).

CHIP, clonal hematopoiesis of indeterminate potential; CI, confidence interval; BMI, body mass index.

**Supplementary Table S4**

Subgroup analysis of risk of new-onset type 2 diabetes based on CHIP status and LDL cholesterol level in patients without medication for dyslipidemia.

|  | New-onset type 2 diabetes  (n = 296) | No event  (n = 3,190) | Adjusted HR (95% CI) | *p* |
| --- | --- | --- | --- | --- |
| Non-hyperLDLC group and non-carrier | 188 | 2184 | 1.00 (reference) |  |
| Non-hyperLDLC group and CHIP carrier | 41 | 414 | 1.00 (0.71–1.41) | 0.995 |
| HyperLDLC group and non-carrier | 52 | 529 | 1.27 (0.93–1.74) | 0.132 |
| HyperLDLC group and CHIP carrier | 15 | 63 | 2.47 (1.46–4.18) | <0.001 |

HR, hazard ratio; CI, confidence interval; CHIP, clonal hematopoiesis of indeterminate potential; BMI, body mass index
